# A Recurrent Episode of Dermatomyositis Associated with Papillary Thyroid Cancer

**DOI:** 10.1155/2017/7985953

**Published:** 2017-07-19

**Authors:** Vijay Gopal Eranki

**Affiliations:** Department of Endocrinology, Swedish American Hospital, 1253 N. Alpine Road, Rockford, IL 61107, USA

## Abstract

**Objective:**

It is uncommon for dermatomyositis to be associated with papillary thyroid cancer. We report an unusual case of papillary thyroid cancer presenting with dermatomyositis.

**Methods:**

The case history, imaging and laboratory data is reviewed.

**Results:**

We report the case of a 62-year-old female with a prior history of dermatomyositis and breast cancer who presented with a recurrent episode of dermatomyositis. Extensive evaluation of the cause of the dermatomyositis recurrence revealed no recurrence of the breast cancer but a thyroid nodule was identified. The nodule was biopsied and the patient was noted to have papillary thyroid cancer. The patient subsequently underwent total thyroidectomy and had gradual improvement in her dermatomyositis.

**Conclusion:**

It is very uncommon for dermatomyositis to be associated with papillary thyroid cancer.

## 1. Introduction

Dermatomyositis is an uncommon condition and occurs in about 1 in 100,000 patients. Based on various studies, dermatomyositis has an association with an underlying malignancy in about 15–25% of the cases [1]. In dermatomyositis associated with a malignancy, breast cancer was noted to be the cause in about 20% of the patients [2]. However, there is very little data on the association of dermatomyositis in association with papillary thyroid cancer. Some of the case reports are from the Far East. Recently, a series of 3 cases identified at the Mayo Clinic over a 40-year period was published. [3]. This suggests the rarity of this condition. Hence, we report the case of a recurrent dermatomyositis associated with papillary thyroid cancer.

## 2. Case Presentation

We present the case of a 62-year-old female patient who presented with dermatomyositis. The patient had a prior known history of breast cancer and associated dermatomyositis about 3 years prior to the current presentation. Her original episode of dermatomyositis was treated with prednisone and hydroxychloroquine. After she received chemotherapy for the treatment of the breast cancer, the rash subsided.

The patient presented to the rheumatologist and oncologist with a recurrence in the dermatomyositis about 2 years after the initial episode. Extensive work-up was done to identify a cause of the recurrence of the dermatomyositis as it is well known that dermatomyositis can be a paraneoplastic syndrome and given that the patient already had an episode of dermatomyositis about 2 years back with breast cancer. The patient even had a bone marrow which was negative for any metastatic disease. The patient had a PET scan which showed uptake in a thyroid nodule. Hence, the patient had a thyroid ultrasound which showed a dominant vascular nodule within the isthmus measuring 1.5 × 1.3 cm with punctate calcifications. There was an adjacent smaller hypoechoic nodule measuring 1.1 cm.

At this point the patient was referred to endocrinology. On detailed history and exam, the patient was noted to have dysphagia and dyspnea as key positive findings. On exam, a palpable mid line thyroid nodule was identified and diffuse rash was noted, especially on bilateral upper extremities and the chest. Also, no obvious neuromuscular deficits were noted on the exam.

## 3. Diagnosis and Treatment

The patient underwent fine needle biopsy of the dominant nodule in the isthmus of the thyroid. The cytology was suspicious for papillary thyroid cancer. In the meantime, the patient was treated by the rheumatologist with prednisone 10–20 mg daily, for the dermatomyositis without much success. The baseline TSH was 4.52 uIU/ml (0.30–4.00).

Finally, the patient underwent a total thyroidectomy a few weeks later. The final pathology showed a unifocal 2.1 cm papillary carcinoma in the isthmus with positive focal margin and indeterminate vascular invasion, and hence it was staged as pT2. Postoperatively when the TSH was 32.29 uIU/ml (0.30–4.00), the thyroglobulin level was 2.3 ng/mL. As the postoperative stimulated thyroglobulin was not significantly high, the radiation oncologist decided not to proceed with radioactive iodine therapy.

## 4. Follow up and Outcome

The patient was started on levothyroxine and dose adjusted to levothyroxine 112 mcg daily. After 4 months, the TSH was 0.552 uIU/ml (0.30–4.00) and the thyroglobulin level was 0.1 ng/mL. At this point the rash had significantly improved on a prednisone taper. The patient recently returned for a follow-up visit and her TSH was 0.218 uIU/ml (0.30–4.00) and her rash completely resolved.

## 5. Discussion

Various studies and case reports have identified the association of dermatomyositis with malignancies. However, there is very limited data on the association of dermatomyositis with papillary thyroid cancer. There are limited case reports from the Far East such as the report from Ikeda et al. in Japan [4] and Ku et al. from Korea [5]. The largest series of 3 cases from Mayo Clinic over a 40-year period indicates the extreme rarity of this condition [3].

The incidence of papillary thyroid cancer is rapidly rising and in 2009 its incidence was noted to be 12.9 per 100,000. [6]. Given the rising incidence of papillary thyroid cancer physicians need to be aware of the possibility of dermatomyositis being associated with the papillary thyroid cancer albeit a rare possibility. Hence, we present this case report to highlight this rare possibility.

Another key finding in our patient is the recurrence episode of the dermatomyositis, both with the breast and with papillary thyroid cancer. This makes our case even more unique and uncommon. Also to our knowledge this is the first case of dermatomyositis with papillary thyroid cancer reported after the 2015 American Thyroid Association Guidelines were published based on which this patient did not have radioactive iodine therapy due to low levels of stimulated thyroglobulin [7].

## 6. Conclusion

Dermatomyositis is an uncommon clinical condition associated in about 15–25% of the cases with an underlying malignancy. However, its association with papillary thyroid cancer is extremely uncommon and very rarely reported. Hence, in patients with refractory dermatomyositis where an extensive malignancy work-up is unyielding, papillary thyroid cancer should also be considered in the differential.
